# Supplementary material for: Boosted output performance of triboelectric nanogenerator via electric double layer effect
Source: Nat Commun. 2016 Oct 5;7:12985. doi: 10.1038/ncomms12985 (PMC5059471; doi:10.1038/ncomms12985)
Supplement: Supplementary Information — Supplementary Figures 1-11 and Supplementary Note 1. [file ncomms12985-s1.pdf]

Supplementary Figures

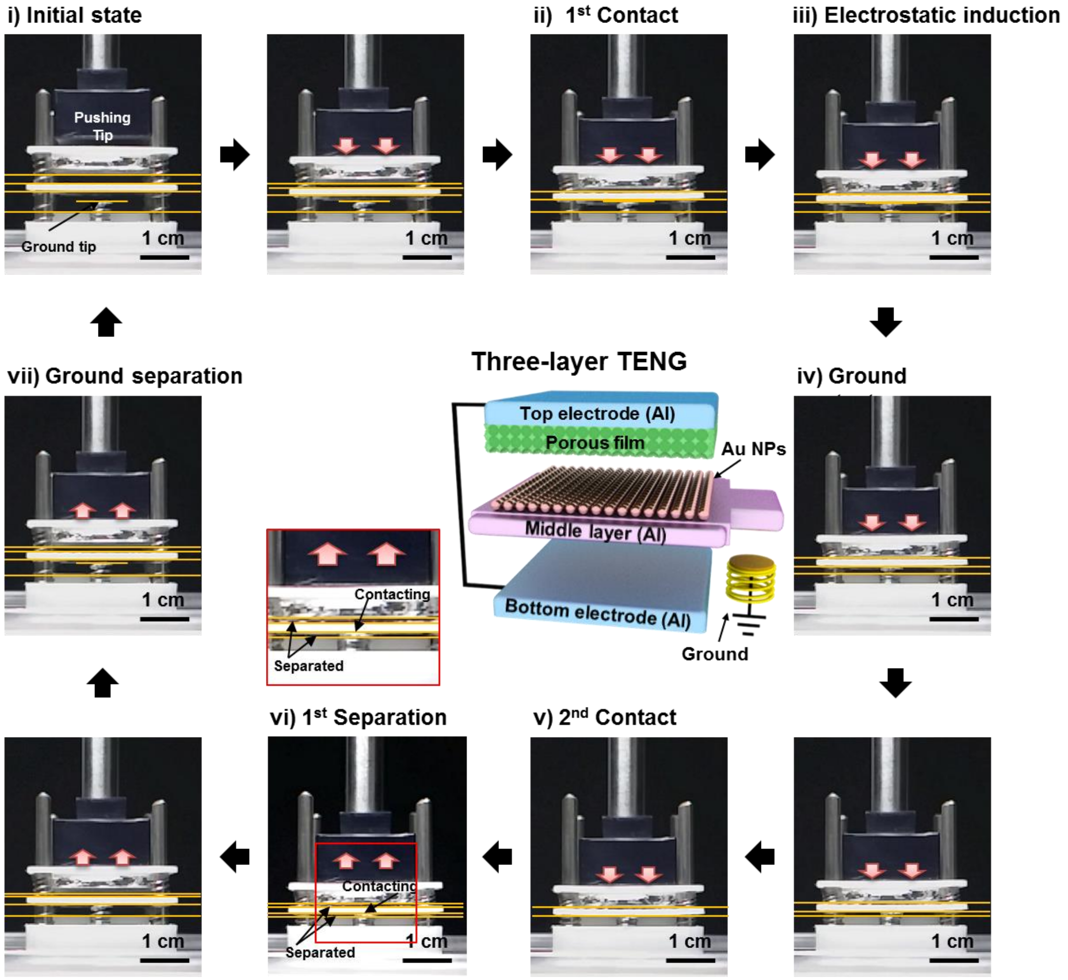

Supplementary Figure 1 | The optical images of the triboelectric nanogenerator when it is pressed and then released.

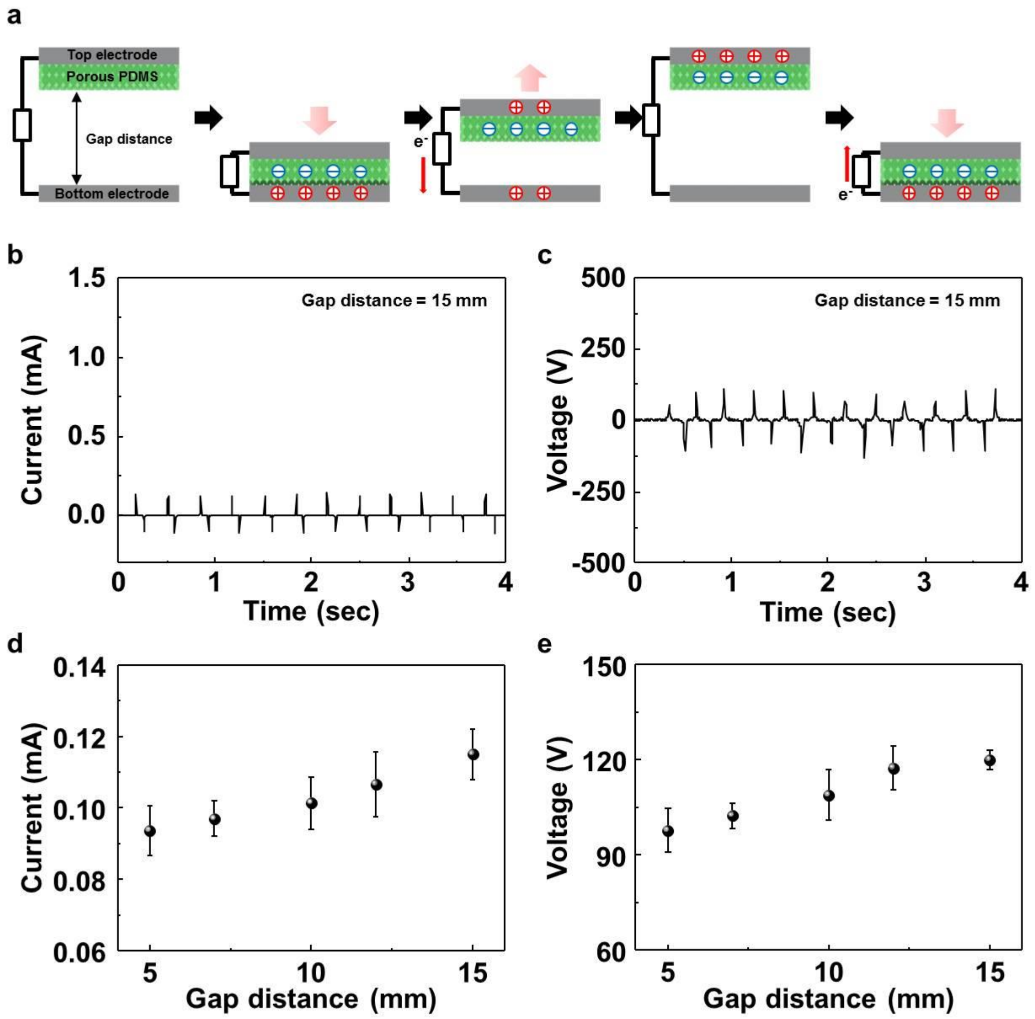

Supplementary Figure 2 | Electrical outputs of two-layer structured triboelectric nanogenerator. (a) Working mechanism for the generation of output voltage and current in the two-layer structured triboelectric nanogenerator under external force. **(b)** The output current and **(c)** voltage of two-layer two-layer structured triboelectric nanogenerator with gap distance of 15 mm. **(d)** The output current and **(e)** voltage of two-layer structured triboelectric nanogenerator with the gap distance of 5, 10, and 15 mm. All error bars in the figure represent s.e.m. of the data.

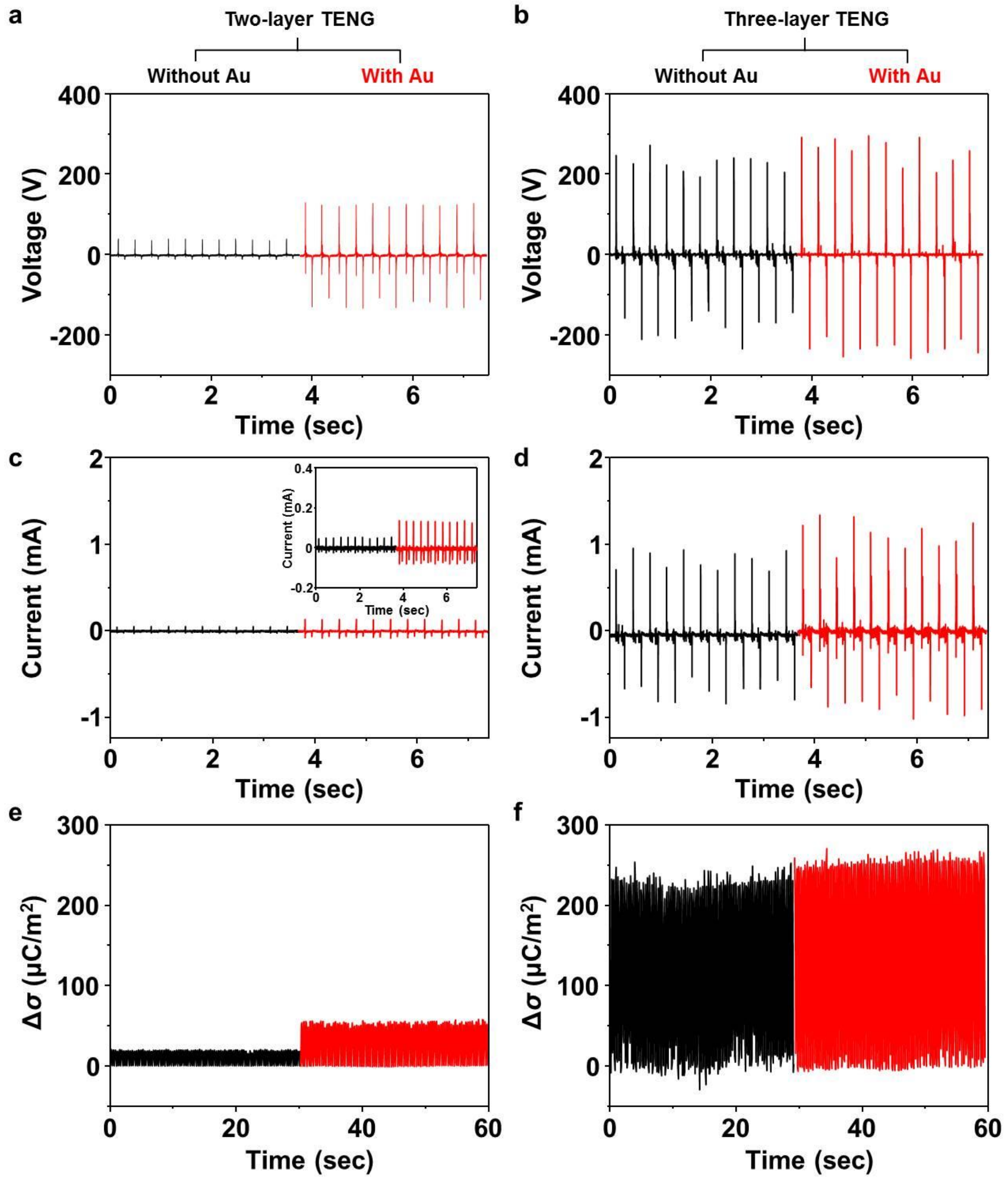

Supplementary Figure 3 | Electrical outputs of triboelectric nanogenerator with and without Au nanoparticles. (a, b) The output voltages, **(c, d)** output currents, and **(e, f)** charge densities of two-layer and three-layer structured TENGs with and without Au nanoparticles.

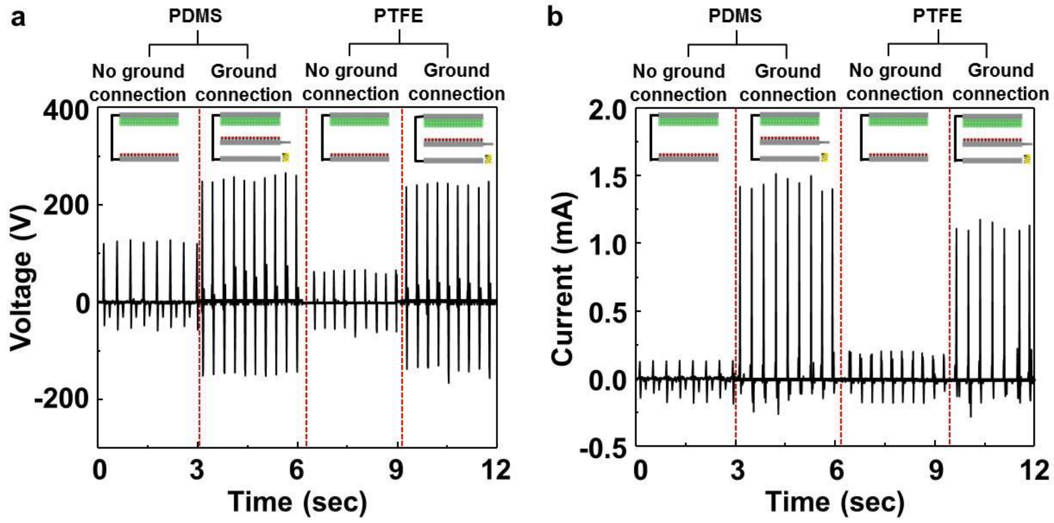

Supplementary Figure 4 | Electrical outputs of three-layer structured triboelectric nanogenerator with various dielectrics. (a) Output voltages and **(b)** currents of three-layer structured triboelectric nanogenerators with Polydimethylsiloxane (PDMS) film and Polytetrafluoroethylene (PTFE) film.

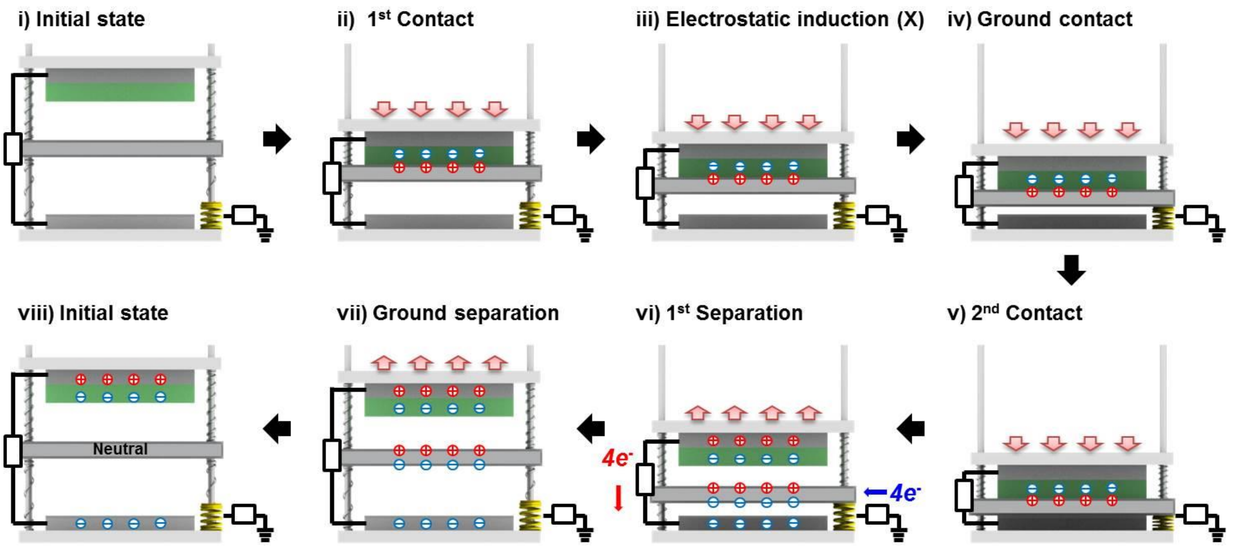

Supplementary Figure 5 | Working mechanism at 1st cycle. Working mechanism for the generation of output voltage and current in the nanogenerator under external force at 1st cycle.

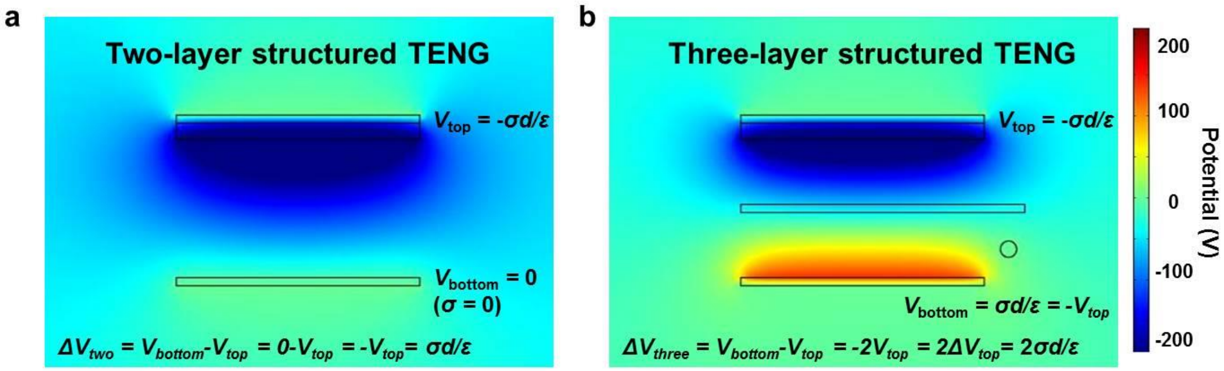

Supplementary Figure 6 | Simulation results. The COMSOL simulations of **(a)** The two-layer structured and **(b)** Three-layer structured triboelectric nanogenerator.

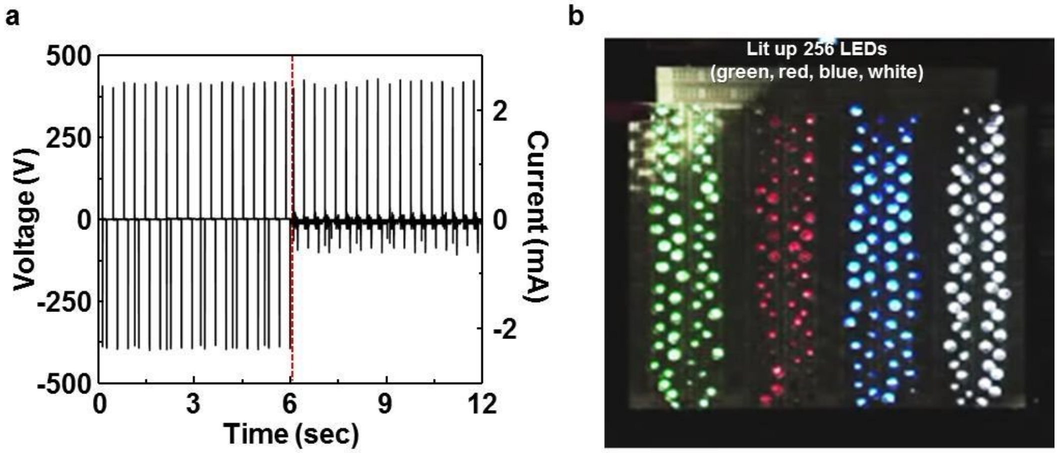

Supplementary Figure 7 | Electrical ouputs of three-layer structured triboeelctric nanogenerator with active area of around 7 × 7 cm^2. (**a**) The output voltage and current of three-layer structured TENGs with active area of around 7 × 7 cm^2. (**b**) The output power from the TENG is able to instantaneously light up 256 LEDs simultaneously.

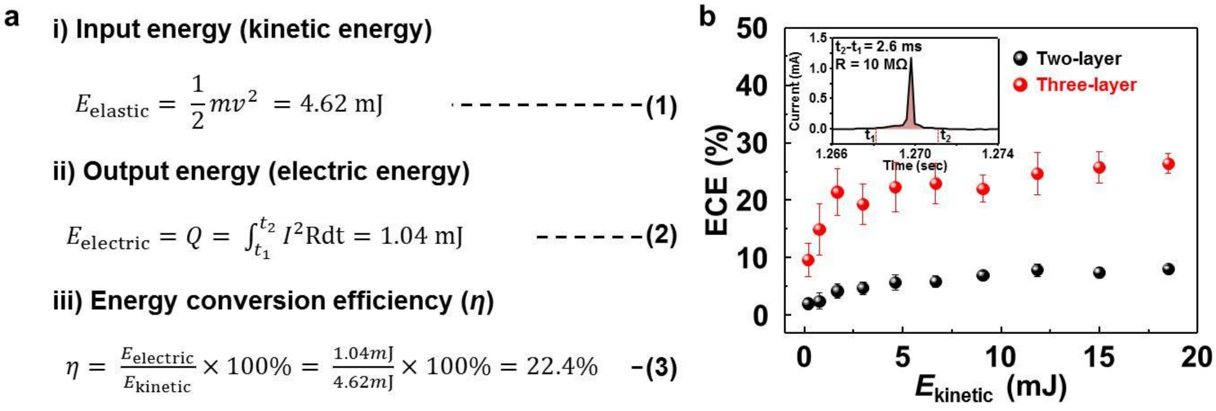

Supplementary Figure 8 | Energy conversion efficiency of the triboelectric nanogenerator. (a) An output current pulse produced by the TENG under same condition. **(b)** The Energy conversion efficiency (ECE, η) change of two- and three-layer structured TENGs as a function of applied kinetic energy from 0.18 to 18.48 mJ. In the three-layer structured TENG. All error bars in the figure represent s.e.m. of the data.

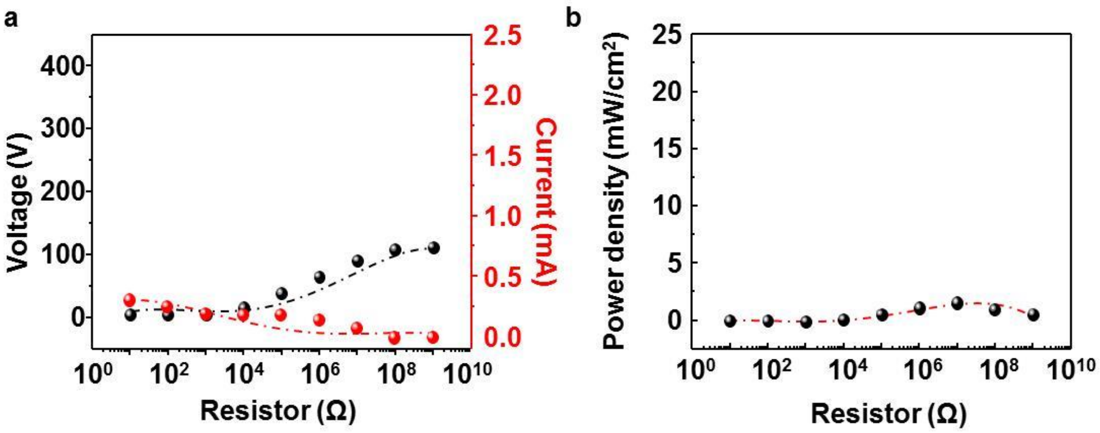

Supplementary Figure 9 | Output power of conventional triboelectric nanogenerator and power density on the surface. (a) The output voltage and current and **(b)** the output power of the conventional TENG with the resistance of external loads from 10 to 10^9 Ω.

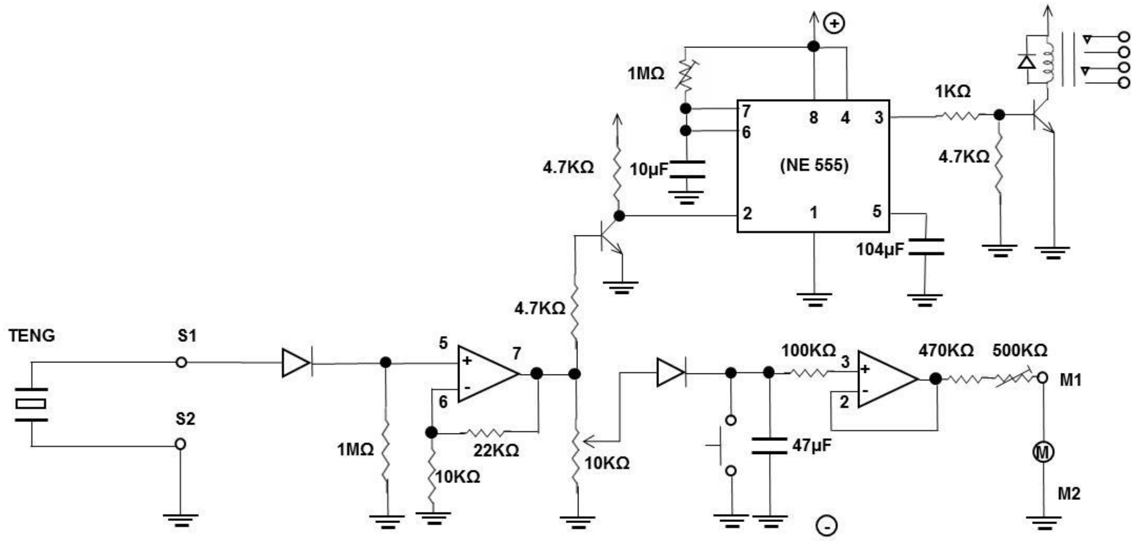

Supplementary Figure 10 | Schematic of AC to DC converting circuit.

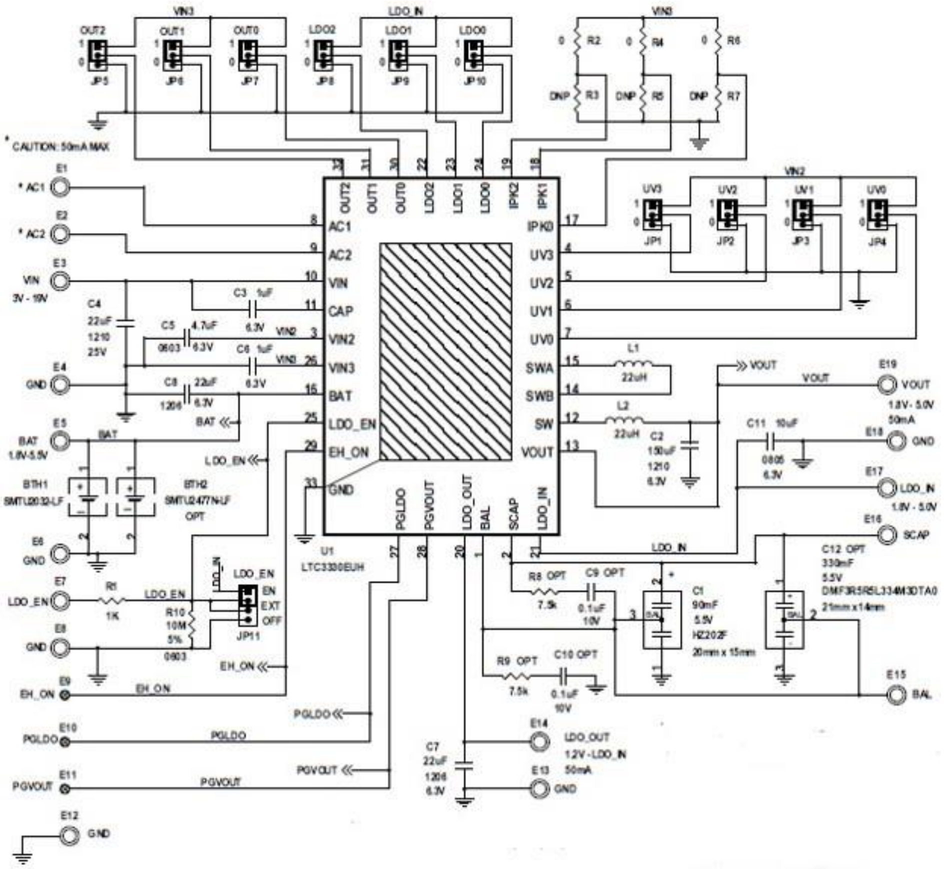

Supplementary Figure 11 | Schematic of buck-boost converter circuit (DC2048A).

Supplementary Notes

Supplementary Note 1. Energy conversion efficiency of the triboelectirc nanogenerator.

When the compressive force of 50 N under a frequency of 3 Hz at a load resistance of 10 MΩ is applied to the top layer, the *v* of the top layer, when a contact is just about to be made, is approximately 0.43 m/s. As an input energy, kinetic energy (E_k) can be defined with the velocity (*v*) and mass (*m*) of top layer, and calculated as below;

$$\mathrm{E_{elastic}} = \frac{1}{2}mv^2 = 4.62\ \mathrm{mJ} \quad (1)$$

Thus, the input kinetic energy can be calculated as 4.62 mJ. Supplementary Fig. 8 shows an output current pulse produced by the TENG under these same conditions. The time span between t_1 and t_2 is 2.6 ms. With an external load of pure resistance, the electric energy delivered by the TENG is equal to the Joule heating energy, as below;

$$\mathrm{E_{electric}} = \mathrm{Q} = \int_{\mathrm{t_1}}^{\mathrm{t_2}} I^2\mathrm{R}dt = 1.04\ \mathrm{mJ} \quad (2)$$

where *Q* is the Joule heating energy, *I* is the instantaneous output current, and *R* is the load resistance. At conversion process level, the elastic energy stored in the springs does not participate in energy conversion. Therefore, the kinetic energy actually converts to electric energy and the ECE (η) at conversion process level is

$$\eta = \frac{\mathrm{E_{electric}}}{\mathrm{E_{kinetic}}} \times 100\% = \frac{1.04\mathrm{mJ}}{4.62\mathrm{mJ}} \times 100\% = 22.4\%\,. \quad (3)$$
